# Metastasectomy or Stereotactic Body Radiation Therapy With or Without Systemic Therapy for Oligometastatic Esophagogastric Cancer

**DOI:** 10.1245/s10434-022-11541-0

**Published:** 2022-04-05

**Authors:** Tiuri E. Kroese, George S. Buijs, Matthijs D. L. Burger, Jelle P. Ruurda, Stella Mook, Lodewijk A. A. Brosens, Peter S. N. van Rossum, Richard van Hillegersberg

**Affiliations:** 1 Department of Surgery, University Medical Center Utrecht, Utrecht University, Utrecht, The Netherlands; 2 Department of Radiation Oncology, University Medical Center Utrecht, Utrecht University, Utrecht, The Netherlands; 3 Department of Pathology, University Medical Center Utrecht, Utrecht University, Utrecht, The Netherlands; 4 Department of Surgery, University Medical Center Utrecht, Heidelberglaan 100, 3584 CX Utrecht, The Netherlands

## Abstract

**Background:**

The primary goal of this study was to determine overall survival (OS) in patients who underwent local treatment (metastasectomy or stereotactic body radiotherapy [SBRT]) or systemic therapy (chemotherapy or targeted therapy) for oligometastatic esophagogastric cancer. The secondary goal was to determine prognostic factors for OS.

**Methods:**

Patients with synchronous or metachronous oligometastatic esophagogastric cancer who underwent local treatment or systemic therapy were included in this single-center, retrospective cohort study. Oligometastatic disease (OMD) included 1 organ or 1 extraregional lymph node station with ≤ 3 lesions. OS was determined after OMD detection. Treatment for OMD was categorized as (1) local treatment, (2) local plus systemic, (3) systemic therapy. The primary tumor was controlled after resection or definitive chemoradiotherapy.

**Results:**

In total, 85 patients were included. Treatment for OMD was local treatment (58%), local plus systemic (14%), or systemic therapy (28%). The primary tumor was controlled in 68% of patients. Most patients were diagnosed with distal esophageal cancer (61%), with adenocarcinoma histology (76%), and presented with synchronous OMD (51%). OS after local treatment was 17 months (95% confidence interval [CI] 12–40), after local plus systemic therapy 35 months (95% CI 29–NA), and after systemic therapy 16 months (95% CI 11–NA). Better OS was independently associated with local plus systemic compared with local treatment (hazard ratio [HR] 2.11, 95% CI 1.05–5.07) or systemic therapy (HR 2.28, 95% CI 1.04–6.07).

**Conclusions:**

Local plus systemic therapy for oligometastatic esophagogastric cancer was independently associated with improved OS and better OS compared with either systemic therapy or local treatment.

**Supplementary Information:**

The online version contains supplementary material available at 10.1245/s10434-022-11541-0.

Gastric and esophageal cancer are the fifth and seventh most common cancer types worldwide with an estimated 1,033,701 and 572,034 new cases annually, respectively.^1^ Between 33 and 50% of esophagogastric cancer patients present with distant metastases at the time of initial diagnosis (i.e., synchronous metastases) and are usually treated with systemic therapy alone or best supportive care (BSC).^2,3^ In addition, between 20 and 50% of patients who have undergone multimodality treatment for locoregional disease develop distant metastases during follow-up (i.e., metachronous metastases).^4–8^

In a small portion of these metastatic patients, distant metastases are present in a limited number of lesions and organs only, so-called oligometastatic disease (OMD).^9^ The concept of OMD was first introduced in 1995 and described a clinical disease state of limited metastatic capacity.^10^ In 2020, a comprehensive classification system for OMD was proposed.^11^ “Induced OMD” was distinguished from “genuine OMD” (i.e., with vs. without previous history of polymetastatic disease, respectively). “Genuine OMD” was subdivided into “repeat OMD” and “de-novo OMD” (i.e., with vs. without a previous history of OMD, respectively).^11^ Finally, “de-novo OMD” was subdivided into “synchronous OMD” and “metachronous OMD.”^11^

Until now, no uniform criteria exist for the maximum number of lesions and organs to be considered OMD in esophagogastric cancer. However, most studies define OMD as a maximum of 3 lesions in 1 organ.^12–14^ Several, small, retrospective, nonrandomized studies suggest that local treatment (i.e., metastasectomy or stereotactic body radiotherapy (SBRT)) for oligometastatic esophagogastric cancer may improve overall survival (OS).^15–19^ However, because these studies have been focusing on patients who underwent local treatment and have not included patients who underwent systemic therapy alone, the general applicability of these studies remains unclear.

Therefore, the primary goal of this study was to determine the OS in patients who underwent local treatment and/or systemic therapy for synchronous or metachronous oligometastatic esophagogastric cancer. The secondary goal was to determine prognostic factors for OS and progression-free survival (PFS).

## Material and methods

### Ethical statement

This study was approved by the Institutional Review Board of the UMC Utrecht, and the need for informed consent was waived. This study was reported in accordance with the STROBE guidelines (Supplementary File 1), The Code of Ethics of the World Medical Association (Declaration of Helsinki) for experiments involving humans, and the Recommendations for the Conduct, Reporting, Editing, and Publication of Scholarly Work in Medical Journals.

### Patient Inclusion

Between 2010 and 2021, consecutive patients diagnosed at the UMC Utrecht with synchronous or metachronous OMD from esophagogastric cancer were eligible for inclusion in this single-center, retrospective cohort study. OMD was limited to 1 organ or 1 extraregional lymph node station with ≤ 3 lesions.^14^ Patients who underwent local treatment and/or systemic therapy for oligometastases were included. Patients who underwent best supportive care were not included. Local treatment was defined as metastasectomy with the intention to perform a radical resection of all metastatic lesions or SBRT of all metastatic lesions using a SBRT scheme: ≥ 10 Gy per fraction with ≥ 1 fraction(s), ≥7 Gy per fraction with ≥ 5 fractions, ≥ 5 Gy per fraction with ≥ 12 fractions, or a total radiation dosage ≥ 50 Gy. Systemic therapy could include targeted therapy and/or chemotherapy.

### Classification of Oligometastatic Lesions

Metachronous OMD was defined as OMD detected after completion of primary tumor treatment (resection of the primary tumor or definitive chemoradiotherapy). The location of OMD lesions was classified into an organ with hematogenous metastasis (i.e., brain, bone, liver, adrenal gland, lung, or soft tissue) or an extraregional lymph node station. OMD lesions were confirmed by pathological assessment or if pathological confirmation was not possible (e.g., because the lesion was not approachable for biopsy) with repeated follow-up imaging. The clinical and pathological stage was classified according to the TNM 8th edition of the International Union Against Cancer (UICC).^20^

### Management

Management for OMD was categorized into (1) local treatment, (2) local plus systemic therapy, or (3) systemic therapy alone. Local treatment consisted of metastasectomy and/or SBRT for OMD. Local plus systemic therapy consisted of local treatment and systemic therapy for OMD. Systemic therapy consisted of systemic therapy alone for OMD. The primary tumor was controlled after resection or definitive chemoradiotherapy, without evidence of loco-regional recurrence.

### Staging

Baseline staging for patients with esophageal cancer was with ^18^fluorodeoxyglucose positron emission tomography (^18^F-FDG PET) with integrated computed tomography (CT).^21^ Baseline staging for patients with gastric cancer was with CT and after 2016 for patients with advanced gastric cancer (i.e., ≥ cT3 or cN+) with PET/CT and diagnostic laparoscopy (according to Dutch national guidelines).^22^ Follow-up was done without standardized imaging and/or endoscopies as recommended in Dutch national guidelines and ESMO and NCCN guidelines).^21–24^ In patients with clinically suspected OMD, ^18^F-FDG PET/CT imaging was performed.

### Outcomes

The primary outcome measure was OS. The secondary outcome measures were prognostic factors for OS and PFS. OS was defined as the time interval between the diagnosis of OMD and death or last follow-up. Prognostic factors for OS were analyzed using multivariable Cox proportional hazard models. PFS was defined as the time interval between the diagnosis of OMD and progression, last follow-up, or death.

### Variables

Performance status was determined at the time of OMD diagnosis according to the World Health Organization (WHO) performance score.^25^ The disease-free interval was defined as the time interval between the detection of the primary tumor and OMD in patients with metachronous OMD.^26^ Recurrence of OMD was categorized into “local” when OMD was detected in the same treated OMD location and “systemic” when OMD was detected in another (nontreated) location. The primary tumor was not controlled in case of locoregional recurrence or no primary tumor treatment.

### Statistical Analysis

Parametric data were presented as mean with standard deviation (SD) and non-parametric as median with interquartile range (IQR). Categorical data were presented as frequencies with proportions. Kaplan-Meier curves were constructed of PFS and OS. Univariable Cox proportional hazard models were used to identify prognostic factors associated with OS. For multivariable analyses, prognostic factors with a *p*-value of <0.25 in univariable analyses were entered in a model, and subsequent backward stepwise elimination was performed according to Akaike Information Criterion.^27^ Prognostic factors were expressed using hazard ratios (HRs) with 95% confidence intervals (CIs). The disease-free interval was dichotomized into ≤ 24 months or > 24 months.^15^ Data were analyzed using R for Windows, version 3.6.3.^28^ A *p*-value < 0.05 was considered statistically significant.

## Results

### Patient Selection

**Fig. 1:**
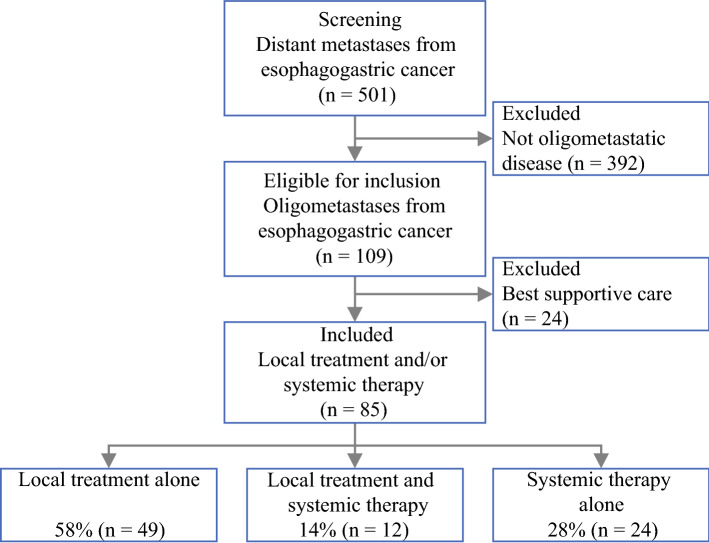
Patient selection

Between 2010 and 2021, a total of 501 patients with synchronous or metachronous distant metastases from esophagogastric cancer were screened for “de-novo” OMD. Synchronous or metachronous OMD was identified in 106 patients (21%). Patients who underwent best supportive care for OMD were not included (*n* = 24). Patients who underwent best supportive care had a worse performance score and more OMD lesions (Supplementary Files 2 and 3). Consequently, a total of 85 “de-novo” OMD patients who underwent local treatment and/or systemic therapy were included. Figure 1 shows the patient inclusion.

### Baseline Characteristics

Included patients had a mean age of 65 years (SD: 9.0), 77% were male, and 91% had a baseline WHO performance score of 0-1. Most patients were diagnosed with esophageal cancer of the distal third of the esophagus (61%) with adenocarcinoma histology (76%) and presented with synchronous OMD (51%). The mean disease-free interval was 24 months (SD: 20) in patients with metachronous OMD. The clinical disease stage was predominantly cT3 (75%) and cN1 (42%). The pathological stage was predominantly pT3 (58%) and pN1 (42%). Table 1 shows the baseline characteristics of included patients stratified for OMD treatment.

**Table 1:** Baseline characteristics stratified for treatment for OMD

Characteristic	Local (*n* = 49)	Local + systemic (*n* = 12)	Systemic (*n* = 24)
Mean age, year [SD]	65.8 (8.7)	60.1 (9.8)	64.6 (8.9)
Sex (%)			
Male	35 (71.4)	10 (83.3)	20 (83.3)
Female	14 (28.6)	2 (16.7)	4 (16.7)
WHO performance score (%)			
WHO 0-1	43 (87.8)	12 (100.0)	22 (91.7)
WHO 2	6 (12.2)	0 (0.0)	2 (8.3)
Location of the primary tumor (%)			
Upper-middle third esophagus	14 (28.6)	1 (8.3)	2 (8.3)
Lower third esophagus	29 (59.2)	9 (75.0)	14 (58.3)
Gastroesophageal junction	6 (12.2)	1 (8.3)	7 (29.2)
Stomach	0 (0.0)	1 (8.3)	1 (4.2)
Clinical tumor stage (%)			
cT1	0 (0.0)	1 (8.3)	0 (0.0)
cT2	3 (6.1)	1 (8.3)	2 (8.3)
cT3	37 (75.5)	7 (58.3)	20 (83.3)
cT4a	6 (12.2)	2 (16.7)	1 (4.2)
Clinical nodal stage (%)			
cN0	10 (20.4)	3 (25.0)	5 (20.8)
cN1	22 (44.9)	3 (25.0)	11 (45.8)
cN2	12 (24.5)	2 (16.7)	2 (8.3)
cN3	3 (6.1)	3 (25.0)	6 (25.0)
Pathological tumor stage (%)*			
pT0	8 (16.3)	1 (8.3)	6 (25.0)
pT1b	3 (6.1)	0 (0.0)	0 (0.0)
pT2	4 (8.2)	0 (0.0)	0 (0.0)
pT3	16 (32.7)	5 (41.7)	2 (8.3)
pT4a	2 (4.1)	2 (16.7)	3 (12.5)
Pathological nodal stage (%)*			
pN0	11 (22.4)	3 (25.0)	6 (25.0)
pN1	15 (30.6)	3 (25.0)	2 (8.3)
pN2	6 (12.2)	0 (0.0)	2 (8.3)
pN3	1 (2.0)	2 (16.7)	0 (0.0)
Histology (%)			
Adenocarcinoma	33 (67.3)	11 (91.7)	21 (87.5)
Squamous cell carcinoma	16 (32.7)	1 (8.3)	3 (12.5)
Signet ring cell carcinoma (%)^†^	3 (6.1)	1 (8.3)	3 (12.5)
Her2neu status positive (%)^†^	10 (20.4)	3 (25.0)	6 (25.0)
Differentiation grade (%)			
Well	5 (10.2)	1 (8.3)	3 (12.5)
Moderate	14 (28.6)	3 (25.0)	11 (45.8)
Poor	27 (55.1)	6 (50.0)	8 (33.3)
Controlled primary tumor	42 (85.7)	9 (75.0)	7 (29.2)
Timing of detection (%)			
Metachronous	23 (46.9)	5 (41.7)	15 (62.5)
Synchronous	26 (53.1)	7 (58.3)	9 (37.5)
Mean disease-free interval, months [SD]*	24.04 (21.97)	28.71 (19.55)	21.33 (18.00)

^*^Metachronous tumors (*n* = 42)

^†^Adenocarcinoma (*n* = 78)

### OMD Characteristics

The location of OMD lesions was the extraregional lymph nodes (28%), liver (21%), bone (21%), brain (15%), lung (8%), adrenal gland (8%), soft tissue (4%), or appendix (1%). The number of OMD lesions was 1 (74%), 2 (21%), or 3 (5%). OMD lesions were confirmed with pathological assessment in 76% or with repeated follow-up imaging in 24%. Most patients with bone, extraregional lymph node, lung, or adrenal gland oligometastases underwent local treatment (62%, 59%, 86%, 57%, respectively). Most patients with liver oligometastases underwent systemic therapy alone (72%). Table 2 demonstrates the location and number of OMD lesions stratified for OMD treatment.

**Table 2:** Location and number of OMD lesions stratified for treatment for OMD

	Local (*n* = 49)		Local + systemic (*n* = 12)		Systemic (*n* = 24)	
Location						
Organ	35	71%	9	75%	17	71%
Liver	3	6%	2	17%	13	54%
Bone	8	16%	3	25%	2	8%
Brain	11	22%	1	8%	0	0%
Adrenal gland	4	8%	2	17%	1	4%
Lung	6	12%	0	0%	1	4%
Soft tissue	2	4%	1	8%	0	0%
Appendix	1	2%	0	0%	0	0%
Extra-regional lymph node	14	29%	3	25%	7	29%
No. lesions						0%
1	41	84%	9	75%	13	54%
2	7	14%	2	17%	9	38%
3	1	2%	1	8%	2	8%
Treatment modalities						
SBRT	30	61%	8	67%	0	0%
Metastasectomy	14	29%	2	17%	0	0%
SBRT + metastasectomy	5	20%	0	0%	0	0%
Chemoradiation therapy	0	0%	2	17%	0	0%
Systemic therapy	0	0%	12	100%	24	100%

### Management for Primary Tumor and OMD

The primary tumor was controlled in 68% of patients. A controlled primary tumor was more common in patients who underwent local treatment or local plus systemic therapy compared with patients who underwent systemic therapy alone (86% and 75% vs. 29%). The primary tumor was controlled after neoadjuvant chemo(radio)therapy followed by resection (51%), chemoradiotherapy (14%), or upfront primary tumor resection (4%).

Treatment for OMD was either “local treatment” (58%), “local plus systemic therapy” (14%), or “systemic therapy” (28%). Local treatment consisted of patients who either underwent SBRT (35% of total), metastasectomy (16%), or metastasectomy and SBRT (6%). Local plus systemic therapy consisted of patients who either underwent systemic therapy plus SBRT (9%), systemic therapy plus metastasectomy (2%), or chemoradiotherapy (2%). Systemic therapy consisted of patients who underwent chemotherapy (21%) or chemotherapy and targeted therapy (7%) as a first-line treatment. Patients with synchronous OMD less often had a controlled primary tumor (42% vs. 76%) and more often underwent systemic therapy alone compared with metachronous OMD (50% vs. 16%). Supplementary Table 4 shows the treatment characteristics stratified for synchronous and metachronous OMD.

### OS and Prognostic Factors

**Fig. 2:**
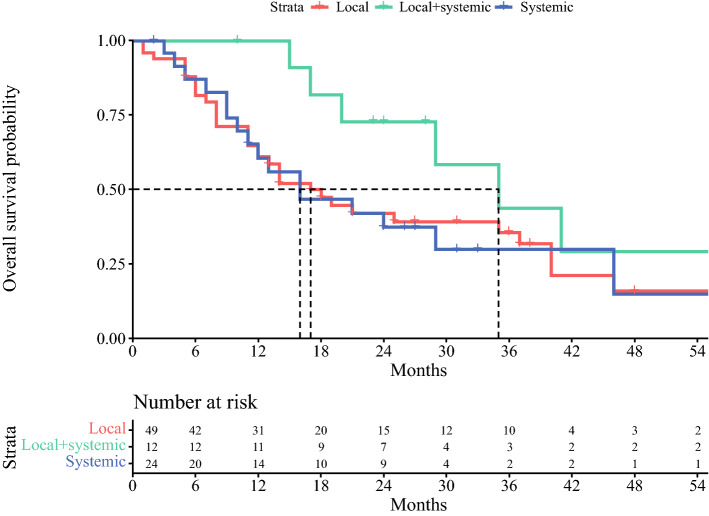
OS stratified for treatment for OMD

Median follow-up time was 17 months (range 1–119). Median OS across all included patients was 20 months (95% CI 15–35). OS after local treatment alone was 17 months (95% CI 12–40), local plus systemic therapy 35 months (95% CI 29–NA), and after systemic therapy alone 16 months (95% CI 11–NA). Figure 2 demonstrates the OS stratified for the treatment for OMD. In multivariable analysis, better OS was independently associated with local and systemic therapy as compared with local treatment alone or systemic therapy alone (HR 2.11, 95% CI 1.05–5.07 and HR 2.28, 95% CI 1.04–6.07). No other prognostic factors for OS were identified in multivariable analyses. Table 3 demonstrates the results of the univariable and multivariable Cox proportional hazard model analyses. OS in the best supportive care group was 6 months (95% CI 4–8, Supplementary File 5).

**Table 3:** Univariable and multivariable Cox proportional hazard models for OS

Characteristic	Overall survival			
	Univariable		Multivariable	
	HR (95% CI)	*p*	HR (95% CI)	*p*
Baseline performance score				
WHO 0	Reference		Reference	
WHO 1-2	1.89 (95% CI 0.85–4.20)	0.116	1.47 (95% CI 0.64–3.36)	0.358
Histology				
Adenocarcinoma	Reference		Reference	
Squamous cell carcinoma	1.89 (95% CI 1.03–3.47)	**0.039**	1.70 (95% CI 0.87–3.30)	0.715
Differentiation grade				
Missing	Reference		Reference	
Well/moderate	1.97 (95% CI 0.58–6.65)	0.229	1.53 (95% CI 0.43–5.45)	0.523
Poor	2.10 (95% CI 0.63–7.09)	0.275	1.51 (95% CI 0.42–5.35)	0.505
Timing of detection				
Synchronous	Reference			
Metachronous	0.85 (95% CI 0.56–1.59)	0.834		
Disease-free interval (mo)				
≤ 24	Reference			
> 24	0.99 (95% CI 0.44––1.89)	0.825		
Location of OMD				
Extraregional lymph nodes	Reference			
Organ	0.89 (95% CI 0.50–1.59)	0.698		
No. OMD lesions*	0.69 (95% CI 0.42–1.15)	0.153	0.70 (95% CI 0.39–1.24)	0.223
Management of OMD				
Local and systemic	Reference		Reference	
Local treatment	2.19 (95% CI 1.03–5.23)	**0.037**	2.11 (95% CI 1.05–5.07)	**0.036**
Systemic therapy	2.21 (95% CI 1.07–5.69)	**0.035**	2.28 (95% CI 1.04–6.07)	**0.034**
Primary tumor				
Not controlled	Reference			
Controlled	0.81 (95% CI 0.46–1.45)	0.496		

Bold values indicate statistical significance

*HR* hazard ratio; *CI* confidence interval; *WHO* World Health Organization

^*^Analyzed as continues variable; Patients with metachronous tumors

### Progression-Free Survival

**Fig. 3:**
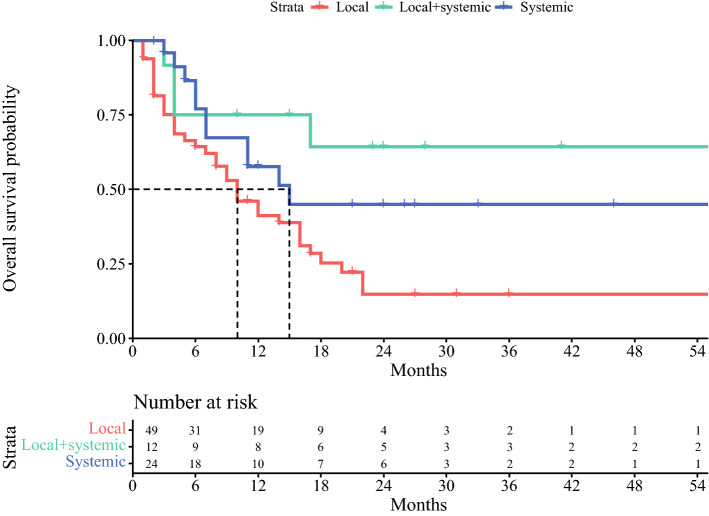
PFS stratified for treatment for OMD

Median PFS across all included patients was 14 months (95% CI 11–21). PFS after local treatment alone was 10 months (95% CI 7–16), local plus systemic median PFS was not reached, and after systemic therapy 15 months (95% CI 11–NA; Fig. 3). A total of 51 patients (60%) developed OMD recurrence during follow-up, of whom 44 patients (51%) developed systemic OMD recurrence and 7 patients (9%) locoregional OMD recurrence (i.e., OMD recurrence in the same treated OMD location).

## Discussion

This cohort study reports on patients with synchronous or metachronous oligometastatic esophagogastric cancer who underwent local treatment (i.e., metastasectomy or SBRT) and/or systemic therapy for OMD. Local treatment combined with systemic therapy for OMD was associated with a favorable OS (median OS 35 months) and independently associated with improved OS compared with patients who underwent local treatment alone (median OS 17 months) or systemic therapy alone (median OS 16 months) for OMD.

This improved OS after local and systemic therapy for OMD compared with local treatment alone might in part be explained by improved PFS (median PFS not reached vs. 10 months) suggesting improved systemic control by adding systemic therapy to local treatment. In addition, this improved OS also might be explained by the selection of patients who responded well to systemic therapy who were offered subsequent local treatment for OMD. Previous studies have administered chemotherapy first as a tool for selecting patients with the highest likelihood to benefit from additional local treatment based on their response to treatment.^29^

OS of patients who underwent local plus systemic therapy was comparable with a phase II, nonrandomized trial that included patients with oligometastatic gastric or gastroesophageal junction cancer patients who underwent systemic therapy and resection of the primary tumor and metastases (35 months versus 31.3 months, respectively).^29^

Moreover, OS patients who underwent local plus systemic therapy for OMD was better than a phase II trial, including patients with esophageal squamous cell carcinoma who underwent SBRT and 50% additional systemic therapy (median OS 24.6).^30^ The lower OS in this trial might be explained by limited use of systemic therapy in this trial (50%). Accordingly, median OS of patients who underwent local treatment alone for OMD *without* systemic therapy in our study was worse as compared with this study (16.0 versus 24.6 months).^30^ Besides the omission of systemic therapy, the lower OS in this cohort might be explained by the inclusion of 12 patients (14% of included patients) with brain OMD in our study (of whom 92% received local treatment), while these patients were excluded from previous trials.^29,30^ Brain OMD are associated with lower OS as compared with extracranial OMD.^31^

Future prospective studies are warranted to determine which patients benefit the most from local treatment for OMD. In our study, OMD was defined as distant metastases in 1 organ or 1 extraregional lymph node station with ≤ 3 lesions. This definition was comparable with recent literature on OMD in esophagogastric cancer.^14^ However, perhaps this definition of OMD should not be applicable to the brain as OMD in this organ is associated with worse OS.^31^ A universal multidisciplinary consensus statement is warranted to initiate future trials in this field. The OligoMetastatic Esophagogastric Cancer (OMEC) consortium was designed to develop a multidisciplinary consensus statement for the definition and treatment for oligometastatic esophagogastric cancer.^14,32^ The OMEC consortium consists of 46 esophagogastric cancer experts centers in Europe and is endorsed by the European Organisation for Research and Treatment of Cancer (EORTC), European Society for Radiotherapy and Oncology (ESTRO), European Society of Medical Oncology (ESMO), European Society of Surgical Oncology (ESSO), European Society for Diseases of the Esophagus (ESDE), the European chapter of the International Gastric Cancer Association (IGCA), and the Dutch Upper GI Cancer Group (DUCG).

There are certain limitations to this study that warrant caution for the interpretation of results. First, selection bias may have resulted in a potential overestimation of OS. Second, the applicability and generalizability of these results remain challenging because of a lack of a uniform definition of OMD in esophagogastric cancer. Strengths of this study include the homogenous study cohort, because only patients with de-novo OMD were included (according to the most recent OMD consensus classification).^11^ Other strengths include the applicability of our results because also patients were included who underwent systemic therapy alone for OMD. Therefore, this study provides a contemporaneous comparator into selection and outcomes after different approaches to treatment for OMD.

## Conclusions

This study included patients with oligometastatic esophagogastric cancer limited to 1 organ with ≤ 3 lesions or 1 extra-regional lymph node station with ≤ 3 lesions who underwent local treatment (metastasectomy or SBRT) and/or systemic therapy for oligometastases. Local treatment combined with systemic therapy was associated with a median OS of 35 months compared with 17 months after local treatment alone and 16 months after systemic therapy alone. Future prospective studies are warranted to confirm these results.

## Supplementary Information

Below is the link to the electronic supplementary material.

Supplementary file1 (DOCX 42 kb)

## Data Availability

All data generated and analyzed during this study are included in this published article (and its supplementary information files).
