# Supplementary material for: Metastasectomy or Stereotactic Body Radiation Therapy With or Without Systemic Therapy for Oligometastatic Esophagogastric Cancer
Source: Ann Surg Oncol. 2022 Apr 5;29(8):4848–57. doi: 10.1245/s10434-022-11541-0 (PMC9246791; doi:10.1245/s10434-022-11541-0)
Supplement: Supplementary file 1 — Supplementary file1 (DOCX 42 kb) [file 10434_2022_11541_MOESM1_ESM.docx]

**Supplementary File 1:** STROBE-checklist

|  | Item No | Recommendation |  |
| --- | --- | --- | --- |
| **Title and abstract** | 1 | (*a*) Indicate the study’s design with a commonly used term in the title or the abstract | Yes |
|  |  | (*b*) Provide in the abstract an informative and balanced summary of what was done and what was found | Yes |
| Introduction | | |  |
| Background/rationale | 2 | Explain the scientific background and rationale for the investigation being reported | Yes |
| Objectives | 3 | State specific objectives, including any prespecified hypotheses | Yes |
| Methods | | |  |
| Study design | 4 | Present key elements of study design early in the paper | Yes |
| Setting | 5 | Describe the setting, locations, and relevant dates, including periods of recruitment, exposure, follow-up, and data collection | Yes |
| Participants | 6 | (*a*) Give the eligibility criteria, and the sources and methods of selection of participants. Describe methods of follow-up | Yes |
|  |  | (*b*) For matched studies, give matching criteria and number of exposed and unexposed | Yes |
| Variables | 7 | Clearly define all outcomes, exposures, predictors, potential confounders, and effect modifiers. Give diagnostic criteria, if applicable | Yes |
| Data sources/ measurement | 8* | For each variable of interest, give sources of data and details of methods of assessment (measurement). Describe comparability of assessment methods if there is more than one group | Yes |
| Bias | 9 | Describe any efforts to address potential sources of bias | Yes |
| Study size | 10 | Explain how the study size was arrived at | NA |
| Quantitative variables | 11 | Explain how quantitative variables were handled in the analyses. If applicable, describe which groupings were chosen and why | Yes |
| Statistical methods | 12 | (*a*) Describe all statistical methods, including those used to control for confounding | Yes |
|  |  | (*b*) Describe any methods used to examine subgroups and interactions | Yes |
|  |  | (*c*) Explain how missing data were addressed | Yes |
|  |  | (*d*) If applicable, explain how loss to follow-up was addressed | Yes |
|  |  | (*e*) Describe any sensitivity analyses | NA |
| Results | | |  |
| Participants | 13* | (a) Report numbers of individuals at each stage of study—eg numbers potentially eligible, examined for eligibility, confirmed eligible, included in the study, completing follow-up, and analysed | Yes |
|  |  | (b) Give reasons for non-participation at each stage | Yes |
|  |  | (c) Consider use of a flow diagram | Yes |
| Descriptive data | 14* | (a) Give characteristics of study participants (eg demographic, clinical, social) and information on exposures and potential confounders | Yes |
|  |  | (b) Indicate number of participants with missing data for each variable of interest | Yes |
|  |  | (c) Summarise follow-up time (eg, average and total amount) | Yes |
| Outcome data | 15* | Report numbers of outcome events or summary measures over time | Yes |
| Main results | 16 | (*a*) Give unadjusted estimates and, if applicable, confounder-adjusted estimates and their precision (eg, 95% confidence interval). Make clear which confounders were adjusted for and why they were included | Yes |
|  |  | (*b*) Report category boundaries when continuous variables were categorized | Yes |
|  |  | (*c*) If relevant, consider translating estimates of relative risk into absolute risk for a meaningful time period | NA |
| Other analyses | 17 | Report other analyses done—eg analyses of subgroups and interactions, and sensitivity analyses | NA |
| Discussion | | |  |
| Key results | 18 | Summarise key results with reference to study objectives | Yes |
| Limitations | 19 | Discuss limitations of the study, taking into account sources of potential bias or imprecision. Discuss both direction and magnitude of any potential bias | Yes |
| Interpretation | 20 | Give a cautious overall interpretation of results considering objectives, limitations, multiplicity of analyses, results from similar studies, and other relevant evidence | Yes |
| Generalisability | 21 | Discuss the generalisability (external validity) of the study results | Yes |
| Other information | | |  |
| Funding | 22 | Give the source of funding and the role of the funders for the present study and, if applicable, for the original study on which the present article is based | 10 |

**Supplementary File 2:** Patient characteristics stratified for BSC

| **Characteristic** | **BSC**  (n = 21) | **Local and/or systemic**  (n = 85**)** |
| --- | --- | --- |
| **Mean age, year [SD]** | 68.57 (10.25) | 64.67 (9.00) |
| **Sex (%)** |  |  |
| Male | 68.57 (10.25) | 64.67 (9.00) |
| Female | 68.57 (10.25) | 64.67 (9.00) |
| **WHO performance score (%)** | | |
| WHO 0-1 | 14 (66.7) | 77 (90.6) |
| WHO 2 | 7 (33.3) | 8 (  9.4) |
| **Location of the primary tumor (%)** | | |
| Upper-middle third esophagus | 3 (14.3) | 17 (20.0) |
| Lowe third esophagus | 13 (61.9) | 52 (61.2) |
| Gastroesophageal junction | 2 ( 9.5) | 14 (16.5) |
| Stomach | 3 (14.3) | 2 (  2.4) |
| **Clinical tumor stage (%)** | |  |
| cT1 | 3 (14.3) | 1 (  1.2) |
| cT3 | 6 (28.6) | 6 (  7.1) |
| cT4 | 9 (42.9) | 64 (75.3) |
| cT4 | 2 ( 9.5) | 9 (10.6) |
| **Clinical nodal stage (%)** | |  |
| cN0 | 11 (52.4) | 18 (21.2) |
| cN1 | 6 (28.6) | 36 (42.4) |
| cN2 | 2 ( 9.5) | 16 (18.8) |
| cN3 | 1 ( 4.8) | 12 (14.1) |
| **Pathological tumor stage (%)*** | | |
| pT0 | 0 (  0.0) | 15 (17.6) |
| pT1b | 3 (14.3) | 3 (  3.5) |
| pT2 | 1 (  4.8) | 4 (  4.7) |
| pT3 | 7 (33.3) | 23 (27.1) |
| pT4a | 2 (  9.5) | 7 (  8.2) |
| **Pathological nodal stage (%)*** |  |  |
| pN0 | 6 (28.6) | 20 (23.5) |
| pN1 | 3 (14.3) | 20 (23.5) |
| pN2 | 1 (  4.8) | 8 (  9.4) |
| pN3 | 3 (14.3) | 3 (  3.5) |
| **Histology (%)** | |  |
| Adenocarcinoma | 16 (76.2) | 65 (76.5) |
| Squamous cell carcinoma | 5 (23.8) | 20 (23.5) |
| **Her2neu status positive (%)†** | 1 (  4.8) | 19 ( 22.4) |
| **Differentiation grade (%)** | |  |
| Well | 3 (14.3) | 9 (10.6) |
| Moderate | 0 (  0.0) | 28 (32.9) |
| Poor | 11 (52.4) | 41 (48.2) |
| **Controlled primary tumor** | 12 (57.1) | 24 (28.2) |
| **Timing of detection (%)** | |  |
| Synchronous | 43 (51%) | 7 (33%) |
| Metachronous | 42 (49%) | 14 (66%) |
| **Mean disease-free interval, months [SD] *** | 13.86 (8.58) | 24.24 (20.47) |
| *= For metachronous tumors; † = For adenocarcinoma) | | |

**Supplementary File 3:** OMD characteristics stratified for BSC

|  | | | **BSC**  (n = 21) | | **Local and/or systemic**  **(**n = 85) | |
| --- | --- | --- | --- | --- | --- | --- |
| **Location** | | |  | |  | |
| Organ | | |  | 0% | 61 | 72% |
| Liver | | | 6 | 29% | 18 | 21% |
| Bone | | | 2 | 10% | 13 | 15% |
| Brain | | | 1 | 5% | 12 | 14% |
| Adrenal gland | | | 0 | 0% | 7 | 8% |
| Lung | | | 4 | 19% | 7 | 8% |
| Soft tissue | | | 2 | 10% | 3 | 4% |
| Appendix | | | 0 | 0% | 1 | 1% |
| Extra-regional lymph node | | | 0 | 0% | 24 | 28% |
| **Number of lesions** | | |  |  |  | |
| 1 | | | 9 | 43% | 63 | 74% |
| 2 | | | 9 | 43% | 18 | 21% |
| 3 | | | 3 | 14% | 4 | 5% |
| **Treatment modalities** | | |  |  |  |  |
| Local | | |  |  |  |  |
| SBRT | | 0 | | 0% | 30 | 35% |
| Metastasectomy | | | 0 | 0% | 14 | 16% |
| Metastasectomy + SBRT | | | 0 | 0% | 5 | 6% |
| Local and systemic | | |  |  |  |  |
| SBRT + systemic therapy | | | 0 | 0% | 8 | 9% |
| Metastasectomy + systemic | | 0 | 0% | 2 | 2% |  |
| Chemoradiation | | 0 | 0% | 2 | 2% |  |
| Systemic therapy alone | | 0 | 0% | 24 | 28% |  |

**Supplementary File 4:** OMD and treatment characteristics stratified for synchronous and metachronous OMD

|  | **Synchronous** | | **Metachronous** | |
| --- | --- | --- | --- | --- |
|  | (n = 43) | | (n = 42) | |
| **Location** |  |  |  |  |
| Organ | 32 | 74% | 39 | 70% |
| Liver | 9 | 21% | 9 | 21% |
| Bone | 10 | 23% | 3 | 7% |
| Brain | 4 | 9% | 8 | 19% |
| Adrenal gland | 5 | 12% | 2 | 5% |
| Lung | 3 | 7% | 4 | 10% |
| Soft tissue | 1 | 2% | 2 | 7% |
| Appendix | 0 | 0% | 1 | 5% |
| Extra-regional lymph node | 11 | 43% | 13 | 30% |
| **Number of OMD lesions** | |  |  |  |
| 1 | 33 | 76% | 30 | 71% |
| 2 | 8 | 19% | 10 | 23% |
| 3 | 2 | 5% | 2 | 5% |
| **Primary tumor treatment** | |  |  |  |
| Controlled | 18 | 42% | 32 | 76% |
| Not controlled | 25 | 58% | 10 | 34% |
| **OMD treatment** | |  |  |  |
| Local | 24 | 55% | 25 | 59% |
| Local and systemic | 6 | 14% | 6 | 13% |
| Systemic | 13 | 30% | 11 | 26% |
|  | **Synchronous** | | **Metachronous** | |
|  | (n = 43) | | (n = 42) | |
| **Location** |  |  |  |  |
| Organ | 32 | 74% | 39 | 70% |
| Liver | 9 | 21% | 9 | 21% |
| Bone | 10 | 23% | 3 | 7% |
| Brain | 4 | 9% | 8 | 19% |
| Adrenal gland | 5 | 12% | 2 | 5% |
| Lung | 3 | 7% | 4 | 10% |
| Soft tissue | 1 | 2% | 2 | 7% |
| Appendix | 0 | 0% | 1 | 5% |
| Extra-regional lymph node | 11 | 43% | 13 | 30% |
| **Number of OMD lesions** | |  |  |  |
| 1 | 33 | 76% | 30 | 71% |
| 2 | 8 | 19% | 10 | 23% |
| 3 | 2 | 5% | 2 | 5% |
| **Primary tumor treatment** | |  |  |  |
| Controlled | 18 | 42% | 32 | 76% |
| Not controlled | 25 | 58% | 10 | 34% |
| **OMD treatment** | |  |  |  |
| Local | 24 | 55% | 25 | 59% |
| Local and systemic | 6 | 14% | 6 | 13% |
| Systemic | 13 | 30% | 11 | 26% |

**Supplementary File 5:** Overall survival curve stratified for BSC.
